# Iontophoresis-assisted transdermal drug delivery for the treatment of inflammatory dermatoses: A review

**DOI:** 10.1016/j.jpha.2025.101512

**Published:** 2025-12-11

**Authors:** Zhixiong Wang, Xiumei Jiang, Changzhao Jiang, Xiaohua Tao, Jincui Ye

**Affiliations:** a Key Laboratory of Neuropsychiatric Drug Research of Zhejiang Province, Institute of Materia Medica, Hangzhou Medical College, Hangzhou, 310013, China; b Zhejiang Provincial People's Hospital, People's Hospital of Hangzhou Medical College, Hangzhou, 310013, China

**Keywords:** Iontophoresis, Inflammatory dermatoses, Transdermal drug delivery, Topical formulations, Synergistic effect

## Abstract

Inflammatory dermatoses, including psoriasis, atopic dermatitis (AD), and acne vulgaris, pose significant challenges in dermatological treatment due to the skin's natural barrier, the stratum corneum (SC), which limits the efficacy of topical formulations. Iontophoresis, a non-invasive technique that applies low-level electrical currents to enhance transdermal drug delivery, has emerged as a promising solution to overcome these barriers. This review explores the application of iontophoresis-assisted drug delivery for treating inflammatory skin conditions, focusing on its mechanisms, benefits, and potential for combination with existing topical formulations. Iontophoresis enhances the penetration of both small molecules, such as corticosteroids, and biological macromolecules, including monoclonal antibodies and nucleic acid-based therapies, into deeper skin layers. This optimized therapeutic delivery maximizes localized efficacy while minimizing systemic exposure and potential adverse effects. Furthermore, when combined with other transdermal enhancement techniques such as microneedles and sonophoresis, iontophoresis demonstrates synergistic effects, facilitating the delivery of challenging drug molecules and enabling controlled, targeted release. Recent advances in wearable iontophoresis devices present new opportunities for continuous and patient-friendly drug administration, particularly for chronic conditions requiring long-term management. Despite these advantages, challenges such as variability in skin response, potential irritation, and device costs remain. Further research and technological advancements are needed to optimize iontophoresis systems for broader clinical applications. Overall, this review highlights the versatility and prospects of iontophoresis as an innovative therapeutic approach in managing inflammatory dermatoses.

## Highlights

• Non-invasive technique: Uses mild electrical currents to drive charged/ionic drugs across the SC via electrorepulsion (ER) and electroosmosis (EO).
• Synergistic combinations: Iontophoresis combined with other techniques significantly boosts efficacy, e.g., chemical enhancers, sonophoresis, and microneedles.
• Innovative designs: Flexible, self-powered systems enable continuous, patient-friendly drug administration.

## Graphical abstract

**Figure:**
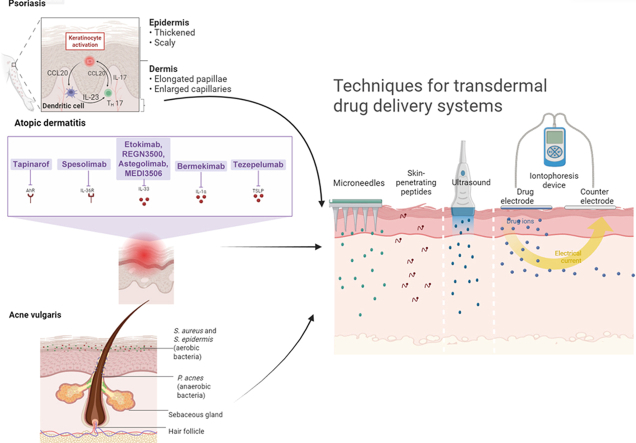

## 1 Introduction

**Fig. 1:**
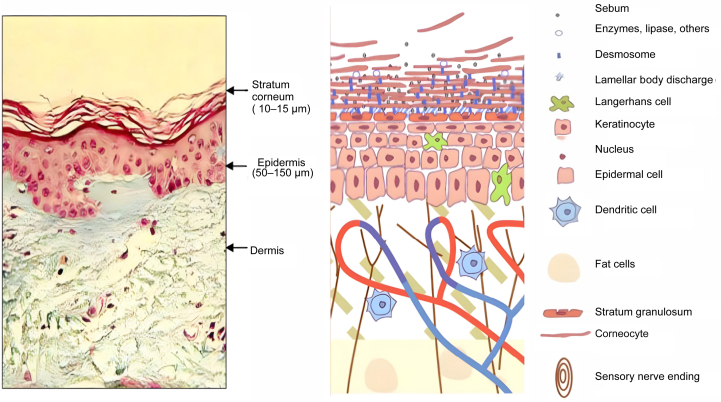
Schematic illustration of the skin structure. Reprinted with permission from Ref. [2].

Inflammatory dermatoses, encompassing a diverse group of chronic conditions, such as psoriasis, eczema, and contact dermatitis, affect millions worldwide, contributing to significant morbidity and decreased quality of life. These disorders often follow a relapsing-remitting course, necessitating long-term management with systemic and topical treatments [1]. The skin is the outermost layer of our body that offers an ample surface area for drug delivery, and comprises two primary layers termed the dermis and epidermis, as is shown in Fig. 1 [2]. Despite the availability of various therapies, the penetration of therapeutic agents remains a major challenge, primarily due to the skin's natural barrier, the stratum corneum (SC). The SC, composed of dead keratinized cells and lipids, is the outermost layer of the epidermis [3]. While this barrier prevents the entry of harmful substances, and hampers the absorption of drugs, this presents a critical challenge in the topical treatment of inflammatory dermatoses, where drugs such as topical corticosteroids (TCSs) and topical calcineurin inhibitors (TCIs) [4] are often administered. Nonetheless, many of these drugs exhibit limited efficacy as their penetration into deeper layers of the skin is often insufficient to achieve therapeutic concentrations at the site of inflammation [5]. Besides, prolonged and frequent use of topical agents may lead to some adverse effects such as skin atrophy, pigmentation changes, and systemic side effects [6]. To address these limitations, alternative methods for enhancing transdermal drug delivery have been explored, with iontophoresis emerging as one of the most promising techniques. Iontophoresis is a non-invasive procedure that applies a low-level electrical current to drive charged or polar molecules through the skin, thereby enhancing drug permeation through the SC [7]. The SC is particularly amenable to iontophoresis due to three important characteristics: a negative background charge, high electrical resistance, and dramatic drops in electrical resistance when the applied voltage is higher than 0.1–2 V [8]. In iontophoresis applications, a mild electric field is applied to increase the mobility of ionized drug molecules across the SC, facilitating deeper penetration and improving therapeutic outcomes [9]. Iontophoresis presents many advantages over other techniques. It is particularly advantageous for treating localized inflammatory conditions, as it facilitates targeted drug delivery with minimal systemic exposure, thereby reducing the risk of side effects commonly associated with systemic treatments. Besides, its painless and non-invasive approach can contribute to increased patient compliance. Table 1 [[10], [11], [12], [13], [14], [15], [16], [17], [18], [19], [20]] briefly summarizes iontophoresis technology in clinical applications. The mechanism of iontophoresis involves two primary processes: electroosmosis (EO) and electrorepulsion (ER). EO refers to the movement of solvent, carrying neutral molecules with it, thereby enhancing the delivery of both non-ionized and ionized drugs. In ER, charged drug molecules are repelled by a similarly charged electrode and driven into the skin [21]. Taken together, these processes facilitate more efficient drug delivery through the SC into deeper layers where inflammation in dermatoses often occurs. Furthermore, iontophoresis offers the advantage of controlled delivery, where the drug delivery rate and the depth of drug penetration can be adjusted by modifying current intensity and duration, which enables personalized treatment tailored to patient needs. Recently, Fukuta et al. [13] investigated the non-invasive delivery of biological macromolecules using iontophoresis, especially for psoriasis treatment. Bozorg et al. [16] demonstrated enhanced hydrocortisone penetration into deeper layers of psoriatic and eczematous skin through iontophoresis compared to passive delivery. Sonaje et al. [22] introduced “iontosomes”, an emerging form of iontophoresis-assisted delivery way for chemotherapeutic drugs. Fukuta et al. [12] explored the synergistic effects of combining iontophoresis with a tight junction-opening peptide to improve the delivery of nucleic acid drugs through thickened psoriatic skin. Wei et al. [23] analyzed the application of iontophoresis in ophthalmology, including its broader applicability for local drug administration use, including anti-inflammatory agents and corticosteroids for eye diseases. These studies collectively highlight iontophoresis as a powerful technique that enhances drug penetration and improves clinical outcomes by alleviating symptoms such as inflammation, itching, and scaling. Besides, iontophoresis holds promise for improving the delivery of large molecules that traditionally face significant challenges in penetrating the skin. Despite its potential, iontophoresis faces several challenges such as skin irritation, variability in individual skin resistance, and the cost of iontophoresis devices. These issues should be addressed before widespread clinical adoption. As research efforts continue to advance, iontophoresis is poised to become a valuable tool in the treatment of inflammatory dermatoses, with enormous potential for integration in wearable devices. This review aims to provide a comprehensive overview of iontophoresis-assisted transdermal drug delivery for the treatment of inflammatory dermatoses. The underlying mechanisms of iontophoresis are examined, followed by an exploration of its applications in specific inflammatory dermatosis conditions. Finally, the challenges and future prospects of this innovative treatment approach are discussed.

**Table 1:** Applications of iontophoresis in dermatological and inflammatory skin conditions.

Application	Drug	Methodology	Outcome	Refs.
Psoriasis (plaque and palmar types)	MTX	Direct current iontophoresis in clinical trials	Enhanced penetration into lesions; similar efficacy to clobetasol; reduced systemic side effects	[10]
Psoriasis	MTX	6-session iontophoresis therapy	The 6 sessions of iontophoresis therapy significantly improved palmoplantar psoriasis, with over 50% of the patients achieving a reduction in lesion severity of more than 50%.	[11]
Psoriasis	NF-κB decoy oligodeoxynucleotides and tight junction-opening peptide AT1002	Iontophoresis with tight-junction opener	Increased skin permeability; effective anti-inflammatory gene therapy in psoriatic skin	[12]
Psoriasis	FITC-labeled IgG antibodies	Iontophoresis in animal models	Enabled intradermal delivery of large biologics; high localization; bypassed systemic delivery issues	[13]
Nail psoriasis	Triamcinolone acetonide	Iontophoresis across nail plate	Improved drug delivery into nail matrix; comparable to daily topical treatment; more convenient	[14]
AD	Cy3-labeled siRNA	Iontophoresis into tape-stripped or sensitized skin	Effective skin delivery and target gene silencing; potential for non-invasive gene therapy	[15]
AD	Hydrocortisone	Iontophoresis into lesional vs. normal skin	Increased drug retention in affected skin layers; superior to passive application	[16]
AD	Tap water	Pulsed current iontophoresis on palms/soles	Reduced severity in some patients; non-pharmacological adjunct to topical therapy	[17]
Acne vulgaris and PIH	APPS, vitamin E	Iontophoresis post-chemical peel	Improved pigmentation, reduced redness and papules; beneficial in scar and PIH treatment	[18]
Acne scars	Tretinoin	Iontophoresis twice per week for 3 months	Reduced scar depth in 93% of patients; also improved skin firmness and pore size	[19]
Eczema/dermatitis	psoralen plus UV A and corticosteroid	Local bath- psoralen plus UV A and iontophoresis	No significant advantage over TCSs; limited additional benefit	[20]

MTX: methotrexate; NF-κB: nuclear factor kappa-B; FITC: fluorescein isothiocyanate; IgG: immunoglobulin G; AD: atopic dermatitis; siRNA: small interfering RNA; APPS: ascorbyl 2-phosphate 6-palmitate; PIH: postinflammatory hyperpigmentation; TCSs: topical corticosteroids; UV: ultraviolet.

## 2 Challenges of conventional transdermal therapy for inflammatory dermatoses

Inflammatory dermatoses, including AD, psoriasis, and contact dermatitis, are chronic, relapsing conditions characterized by complex interplay of immune dysregulation, skin barrier dysfunction, and genetic susceptibility. These diseases impose a substantial burden on patient's quality of life and often require long-term pharmacological management. Transdermal drug delivery is widely used in dermatology due to its inherent advantages, encompassing localized action, reduced systemic toxicity, and improved patient compliance. However, traditional topical approaches relying on passive diffusion through the skin encounter significant physiological and pharmacological limitations, especially when applied to severe disease, hyperkeratotic lesions, or when attempting to deliver macromolecular biologics [24].

### 2.1 Impaired skin barrier and poor drug distribution

The SC, made of corneocytes within a lipid matrix, forms a major barrier to transdermal drug delivery by severely limiting the passive diffusion of most therapeutic agents. In psoriasis, the SC is abnormally thickened due to hyperkeratosis and parakeratosis, which further impedes drug penetration. Calcipotriol, a vitamin D analog used in psoriasis, exhibits markedly reduced efficacy when applied to hyperkeratotic plaques unless formulated with a keratolytic agent like salicylic acid [25]. Conversely, atopic dermatitis (AD) involves a compromised barrier due to factors such as filaggrin mutations, ceramide deficiency, and disrupted tight junctions. However, this barrier does not translate to improved drug delivery. Hydrophilic drugs such as tacrolimus still demonstrate poor permeability and may provoke local burning and stinging, especially during disease flares [26]. To compensate for the limited penetration of therapeutic agents, high-potency corticosteroids are often used. However, this approach raises the risk of cutaneous adverse effects (atrophy, telangiectasia, and striae) and systemic absorption, particularly when used under occlusion or for prolonged durations [27]. Even after a drug successfully permeates the SC, its distribution within inflamed skin is highly heterogeneous. The unique microenvironment of inflammatory lesions, characterized by altered vascularization, irregular epidermal turnover, edema, and barrier heterogeneity, significantly complicates drug diffusion and retention [28]. In conditions such as plaque psoriasis, areas of thick plaque may receive subtherapeutic concentrations of topicals, while thinner or intertriginous areas absorb a greater amount of the drug, thereby raising the risk of localized side effects. For instance, betamethasone dipropionate cream applied to both the elbows and the groin yields insufficient efficacy but induces skin thinning at the groin [29]. TCSs, even when applied at standard doses, have been associated with hypothalamic-pituitary-adrenal (HPA) axis suppression, especially in children or when used on large body surface areas or under occlusion. A classic case involves hydrocortisone 2.5% cream leading to measurable cortisol suppression in infants treated for diaper dermatitis [30]. Disease-related variability also affects absorption kinetics. In acute eczema, where the skin is exudative and inflamed, drug absorption may be rapid and unpredictable. In contrast, lichenified lesions exhibit poor penetration, leading to reduced efficacy [16]. To address these challenges, iontophoresis offers a promising solution. It effectively bypasses the SC by actively delivering charged or hydrophilic drugs into deeper skin layers, providing a controlled and targeted approach for treating inflammatory dermatoses.

### 2.2 Limited applicability to large macromolecular drugs

The advent of biologic therapies, particularly monoclonal antibodies targeting tumor necrosis factor-alpha (TNF-α), interleukin-17 (IL-17), IL-12/23, and IL-4/13, has revolutionized the therapeutic landscape for moderate-to-severe inflammatory dermatoses. However, these agents typically have molecular weights between 140 and 160 kDa and their hydrophilic nature renders them entirely incompatible with passive transdermal delivery [31]. Secukinumab (anti-IL-17A, 150 kDa), adalimumab (anti-TNF-α, 148 kDa), and ustekinumab (anti-IL-12/23, 148 kDa) are highly effective in treating psoriasis and AD, yet require subcutaneous injection. This administration route introduces barriers such as needle anxiety, pain, risk of injection-site reactions, and higher costs associated with refrigeration and the need for clinical supervision [32]. Moreover, patients with mild or moderate disease may benefit from biologic-level efficacy but often cannot access these drugs since their injection-only formats are deemed excessive or impractical for less severe conditions. This creates a distinct therapeutic gap between topicals and systemic injectables. While early-stage research into topical antibody fragments, nanocarriers, and iontophoresis-enhanced delivery shows promise, these technologies are still in development and not yet clinically available [33].

## 3 Mechanisms and influencing factors of iontophoretic transport

**Fig. 2:**
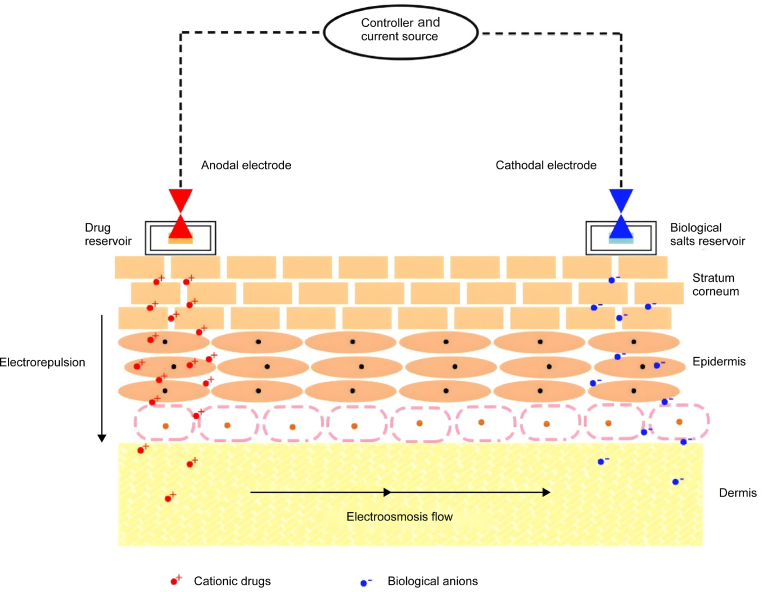
A schematic diagram of iontophoretic technique. Electrorepulsion (ER) involves the repulsion of the drug cations and the movements through the skin, while electroosmosis (EO) involves the movements towards the cathode. Reprinted with permission from Ref. [7].

An iontophoresis system typically comprises a positive electrode anode and a negative electrode cathode, a microprocessor, a drug reservoir, and a power source (Fig. 2) [7]. When the active electrode containing the drug formulation is applied to the skin, the circuit is completed via a "return" electrode placed adjacent to it [34]. The applied electrical current then drives drug permeation through the skin. This method is particularly well-suited for the delivery of hydrophilic, charged low-molecular-weight molecules. The current amplitude is adjustable for each application but is typically <0.5 mA/cm^2^ to avoid adverse effects, ensuring physiological compatibility [35].

According to Faraday's law, the steady-state iontophoretic flux of a charged species, X results from two distinct transport mechanisms: electromigration *J*_EM,X_ (ER) and EO *J*_EO,X_. ER arises from the direct effect of an applied electric field on charged entities, driven by Coulombic repulsion between electrodes and charged drugs. It directs the transport of charged molecules across the skin, including trans-follicular migration. During this process, electron flux converts to ionic flux via electrode reactions, with ionic transport across biological membranes maintaining electroneutrality. Electromigration follows Faraday's law, expressed as $J_{X}^{EM}=\frac{1}{z_{X}AF}\cdot t_{X}I$, where $t_{X}$ is the transport number of species *X* (the fraction of total current carried by ion X), I is the total current applied, $z_{X}$ is the valence (charge number) of species *X*, F is Faraday's constant, and A is the electrode area through which the current is applied [35,36]. Electromigration typically dominates for small ions, although its efficiency declines with molecular weight, and is influenced by drug charge and competing ions. For example, the transport number of biomolecules like immunoglobulin G can decrease substantially. Besides, molecules with a higher valence experience stronger electromigrative forces and therefore exhibit greater flux [37]. Conversely, the presence of competing ions in the formulation can reduce delivery efficiency. For instance, in solutions with high sodium chloride concentrations, the abundance of Na^+^ competes with lidocaine for current, reducing lidocaine's transport number and cutting its iontophoretic flux [38]. In contrast, EO involves the bulk movement of solvent that carries both ions and neutral molecules along with the flow, aligning with counterion movement across membranes. Driven by the net skin charge (isoelectric point approximately 4–4.5), this phenomenon generates positive counterions that propel solvent flow from anode to cathode but hinder cathodic delivery [39]. EO follows the equation: $J_{X}^{EO}=vc_{X}$, where v is the electroosmotic solvent velocity, and $c_{X}$ is the concentration of the drug. EO is particularly important for neutral molecules like glucose and large cationic molecules (>10 kDa), contributing most of their total flux, especially when compared with small ions. This process is also strongly modulated by both pH and ionic strength of the delivery medium [40].

While theoretical equations suggest linear flux-current density correlations, experimental findings have shown inconsistencies. Kalaria et al. [41] observed no strict linear flux-concentration relationship for rasagiline (RAS) and pramipexole. Patel et al. [42] found tacrine delivery increased with low concentrations but plateaued at higher ones. Djabri et al. [43] reported that midazolam iontophoresis markedly increased transport across intact porcine skin compared to passive delivery. At 6 h, the flux was 14 ± 0.4 pmol/h without current, compared to 2.4 ± 0.64, 5.4 ± 0.57, and 10.4 ± 3.8 nmol/h at 0.1, 0.2, and 0.36 mA, respectively. Conversely, Kalaria et al. [44] showed current density linearly increased flux, resulting in linear steady-state flux across human/porcine skin. Taken together, these findings indicate that the effects of current density on flux vary with experimental conditions, formulations, and drug physicochemical properties.

While higher drug concentrations typically increase iontophoretic flux, the relationship is not always linear, complicated by drug-membrane interactions, drug physicochemical properties, and system conditions. The presence of competing ions is particularly critical since the use of salt bridges to eliminate competing cations in the donor compartment can make drug delivery concentration-independent [45]. For example, a 7-h iontophoresis of RAS with a salt bridge (0.5 mA/cm^2^) showed equivalent cumulative permeation across 10, 20, and 40 mM (1200.4 ± 154.6, 1262.6 ± 125.0, 1321.5 ± 335.2 μg/cm^2^) [44]. Electrodes facilitate charged drug transport via current, with the material choice impacting efficiency, stability, and skin irritation, with various materials presenting context-dependent pros and cons [7]. While a wide range of electrode materials can be utilized in iontophoresis, Ag/AgCl electrodes are favored due to their capacity to mitigate pH decreases. Moreover, their electrochemical reactions proceed at a lower voltage than that required to initiate water electrolysis. Since water electrolysis generates protons that can compete with cationic drugs for the applied current, diminishing the efficiency of drug delivery, the use of Ag/AgCl electrodes, which circumvent this process provides a substantial advantage in preserving drug transport efficacy [46]. The pH of the drug solution is also crucial for iontophoretic delivery, primarily influencing drug ionization: for weak bases, a higher pH reduces ionization, thereby diminishing ER. Asma Djabri et al. [43] found iontophoretic transport increased with pH to some extent: the J_6_h was 18 ± 3.2 nmol/h at pH 4.5 (significantly higher, *P* < 0.05, than 6.0 ± 3.0 nmol/h at pH 3). At pH 4.5, midazolam maintained balanced ionization for optimal transport, with less proton competition than at pH 3, thereby enhancing ER efficiency.

## 4 Iontophoresis combined with other methods for transdermal drug delivery systems (TDDS) enhancement

As the skin inherently presents a barrier to the penetration and delivery of drugs, physical and chemical methods have been explored to achieve synergistic effects for iontophoretic drug delivery. Therefore, in this section, we explore the potential of the iontophoresis technique to enhance the transdermal delivery of therapeutic agents combined with other techniques by further overcoming the SC barrier.

### 4.1 Iontophoresis assisted drug delivery with chemical enhancers

The SC, with its corneocytes surrounded by lipid layers, acts as a natural barrier to percutaneous absorption, thereby preventing the electric current's ability to deliver drugs to the targeted site. A study conducted by Choi et al. [47] measured the blood glucose level after iontophoretic insulin delivery combined with chemical enhancers in rabbits. Ethanol (EtOH), propylene glycol (PG), and PG:EtOH (7:3, *v/v*) were selected as solvents, alongside oleic acid (OA) and linoleic acid as chemical enhancers to identify synergistic drug delivery effects. Rabbits pretreated with 0.2 M OA (PG) for 1 h and 0.3 M OA (PG) for 30 min groups exhibited a significant decline in blood glucose. In contrast, no significant change was observed in rabbits pretreated with 0.1M LA (EtOH) for 30 min and in control rabbits. These findings suggest that chemical enhancer pretreatment for iontophoresis use can reduce electrical impedance, thereby facilitating drug delivery and holding promise for future research. Another study by Kanikkannan et al*.* [48] investigated the effect of skin pretreatment with Azone® (laurocapram) in conjunction with iontophoresis on the pharmacodynamic effect of timolol maleate (TM). The results revealed that the onset of action for iontophoretically delivered TM (1.0 mg/mL) was comparable to that of intravenous administration. Furthermore, the $E_{\max}$ and duration of the effect and the area under the curve (AUC) for iontophoretically delivered TM (1.0 mg/mL) were higher than those achieved with intravenously administered TM. These two studies collectively illustrate that the combined use of iontophoresis and chemical enhancers can substantially reduce the drug concentration required to achieve desired therapeutic effects and significantly enhance transdermal drug penetration compared with iontophoresis alone.

### 4.2 Iontophoresis-assisted drug delivery with sonophoresis

Sonophoresis, also known as ultrasound or phonophoresis, is characterized by the transport of drugs through the skin and into soft tissue following the influence of an ultrasonic perturbation [49]. Sonophoresis is a non-invasive method that facilitates the delivery of both hydrophilic and lipophilic compounds, thereby expanding its applicability for a broader range of therapeutic agents. Besides, sonophoresis offers high precision, allowing for the optimization of drug delivery by adjustments in ultrasound frequency and intensity. Conversely, sonophoresis has limitations that should be acknowledged. The potential for localized overheating or tissue damage poses a risk if procedures are not properly managed, which could reduce patient comfort and acceptance in certain clinical scenarios. Operational complexity can also hinder ease of use, necessitating specialized equipment and trained personnel for effective application [50].

**Fig. 3:**
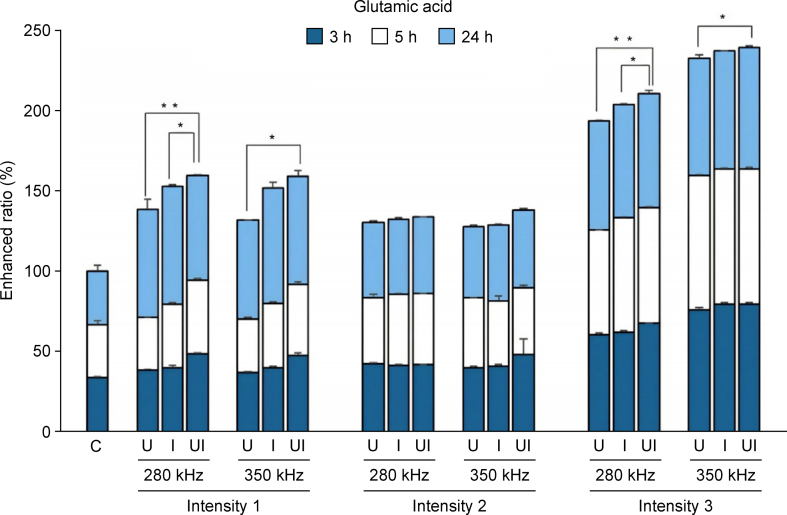
Enhancement ratio of the glutamic acid on skin at different frequencies (280 and 350 kHz) and intensities (levels 1, 2, and 3). Reprinted with permission from Ref. [53]. U: only ultrasound treatment; I: only iontophoresis treatment; UI: the simultaneous treatment of ultrasound and iontophoresis.

Iontophoresis uses electric fields to enhance the transdermal delivery of charged drugs with precise control over dosage by adjusting current levels. It improves bioavailability by avoiding gastrointestinal degradation and first-pass metabolism. Nonetheless, the requirement for electrical equipment can lead to discomfort due to skin irritation, which may reduce patient compliance over time. The efficacy of iontophoresis is also contingent upon the drug's ionic characteristics, limiting its use to ionizable substances and complicating the treatment of non-ionic compounds [51]. This synergistic approach is particularly effective for macromolecules, such as peptide dendrimers, which are challenging to deliver transdermally using conventional methods. Sonophoresis increases permeability to large molecules, while iontophoresis provides controlled transport through those enhanced channels. Adjusting ultrasonic parameters enables precise dosing, reduces side effects such as plasma spikes commonly associated with systemic delivery, and enhances patient compliance. This combination broadens applicability to a wide range of drugs, from small molecules to biologics, including those with poor passive diffusion, such as hydrophilic compounds used in pain and cancer therapies [52]. It has been reported that the synergistic application of iontophoresis and sonophoresis showed improved permeation of cosmeceutical drugs such as niacinamide and retinol. This combination allows for shorter treatment times and reduced skin irritation. A study conducted by Park et al. [53] investigated the synergistic application of sonophoresis and iontophoresis on skin penetration for delivery of hydrophilic and hydrophobic drugs. The experiment was performed at various intensities and frequencies with single and simultaneous application of the two techniques. The improvement observed in all treatments groups was statistically significant compared with the control group. Owing to the negative charge and ionic property of glutamic acid, iontophoresis exhibited a more pronounced effect on enhancing skin penetration in this experiment (Fig. 3) [53]. Taken together, previous studies suggest that simultaneous application of iontophoresis and sonophoresis produces a more enhanced effect compared to individual application if either iontophoresis or sonophoresis.

### 4.3 Iontophoresis-assisted drug delivery with microneedles

The microneedle technique involves the creation of microscale punctures in the SC, which enhances the permeability of drug molecules across this barrier. This approach also avoids gastrointestinal degradation and hepatic first-pass metabolism, thereby reducing systemic drug exposure and minimizing systemic side effects [54]. Compared to conventional needle injections, this technique is associated with reduced pain and discomfort, thereby promoting improved patient compliance with treatment protocols. Furthermore, microneedles offer the benefit of self-administration, which is particularly advantageous in public health initiatives, such as vaccination campaigns [55]. However, microneedles also present certain challenges. Variability in penetration depth and efficiency can occur depending on the individual's skin type, potentially leading to inconsistent drug delivery. Besides, issues related to manufacturing and cost, along with the need for precise application techniques, can hinder widespread clinical adoption [56].

The combination of microneedles with iontophoresis provides a synergistic approach for enhancing transdermal delivery. Microneedles create microchannels that effectively bypass the SC, allowing iontophoresis to more effectively drive charged drugs through the skin. This results in significantly higher drug delivery rates than using either method alone, for example, with drugs like tofacitinib [57]. The combination enhances drug flux, especially for ionic compounds, and ensures more consistent delivery profiles, while maintaining a minimally invasive and largely painless delivery process, thereby promoting better patient compliance. Moreover, it enables the development of user-friendly, self-administrable systems, which are ideal for mass immunization campaigns and chronic disease management. When used in combination with iontophoresis, drug permeation is enhanced significantly. Noh et al. [57] designed a study to investigate the possibility of transdermal delivery of macromolecules, specifically recombinant human growth hormone (rhGH) by combining iontophoresis and a novel microneedle device (Tappy Tok Tok®). The group that received microneedle pre-treatment followed by optimized iontophoresis showed a cumulative permeation that was 6.73 times higher than the group treated with iontophoresis alone, and 1.92 times higher than the group treated with microneedle pre-treatment alone. The synergetic effect of microneedle application and iontophoresis on permeation was also clearly observed in steady-state flux [57,58]. Taken together, the synergistic application of microneedles and iontophoresis yields a markedly superior permeation-enhancing effect compared to the individual use of either technique.

**Fig. 4:**
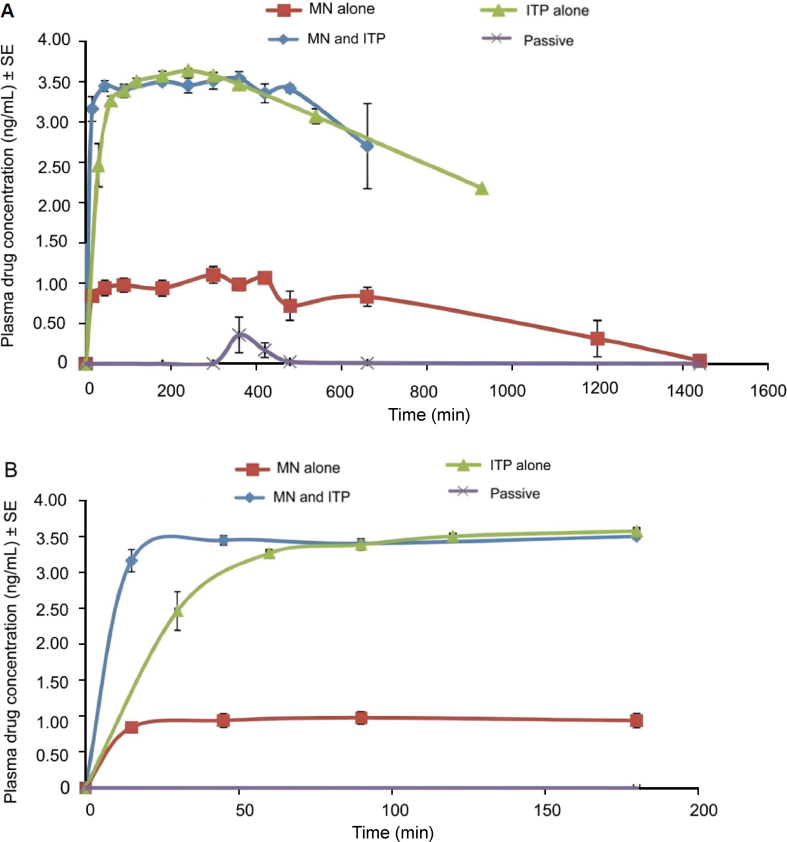
Comparison of transdermal delivery methods for leuprolide acetate. (A) Plasma levels of leuprolide acetate obtained following *in vivo* transdermal delivery in hairless rats by passive diffusion as control (cross), through skin pretreated with three-layer 500 m long maltose microneedles (MN; square), through skin by iontophoresis (ITP) alone at 0.1 mA/cm^2^ (triangle) and through skin by combination of ITP at 0.1 mA/cm^2^ and microneedles treatments (diamond). (B) Magnified image of the initial part of plasma profile of leuprolide acetate achieved by the four treatments. The error bars indicate standard error (SE). Reprinted with permission from Ref. [59].

Another study conducted by Sachdeva et al. [59] investigated the application of iontophoresis and microneedles to enhance transdermal delivery of leuprolide acetate *in vivo* in hairless rats. Their delivery studies were conducted using iontophoresis alone, microneedles alone, and a combination of both. Analysis of variance (ANOVA) demonstrated a statistically significant enhancement in drug delivery with the combination treatment, yielding substantially higher drug levels compared to microneedle-only and passive treatments (*P* < 0.05), as shown in Fig. 4 [59]. However, further analysis using Tukey's test illustrated no significant difference between the iontophoresis-alone and the combination treatment groups. The rationale was subsequently elucidated. Existing evidence confirms that the electrotransport of drug molecules is fundamentally governed by two key mechanisms: electro-repulsion and electro-osmosis [60]. The formation of micropores in the skin is widely believed to significantly enhance iontophoretic delivery, where ER serves as the primary mechanism facilitating molecular transport. In this study, however, electro-osmosis served as the dominant mechanism for drug delivery, with the process relying on solvent drag that occurs primarily through the appendageal routes [59]. In summary, the application of the iontophoretic transdermal drug delivery combined with microneedles in most cases can substantially enhance drug delivery compared to iontophoresis or microneedles used alone. In contrast, the outcomes may be profoundly impacted by the fundamental mechanisms governing drug delivery under applied current, the inherent physicochemical properties of the permeant, and the precise experimental conditions utilized.

## 5 Potential for the applications of iontophoresis in wearable devices

Wearable technologies enable seamless, continuous monitoring and data collection throughout the day, providing advanced, dynamic, and intelligent analyses of multiple health parameters. These capabilities not only enhance the medical management of chronic conditions but also enable remote disease surveillance, personalized adjustment of treatment regimens, and lifestyle optimization through integration with cloud-based platforms. This approach represents a pivotal advancement in the proactive control and management of chronic diseases [61,62]. The convergence of iontophoresis with wearable technologies represents a transformative leap in personalized medicine, enabling closed-loop diagnosis-therapy systems that bridge real-time physiological monitoring with precision drug delivery. This integrated approach directly addresses the limitations of traditional transdermal methods by leveraging advancements in flexible electronics, multimodal sensing, and intelligent algorithms, as evidenced by recent breakthroughs in bioelectronic engineering. Below is an expanded analysis integrating deeper technical insights, clinical validations, and emerging trends.

### 5.1 Advanced iontophoresis platforms: innovation, integration, and regulatory complexity

**Fig. 5:**
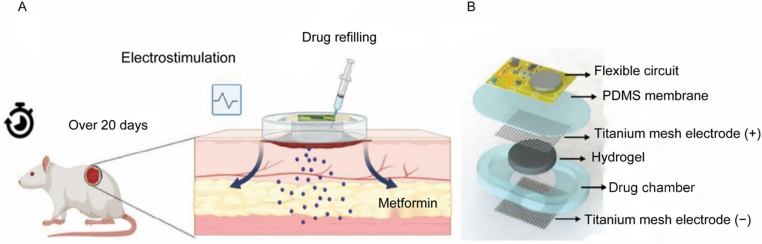
Hydrogel bioelectronics for programmable drug delivery. (A) Functional schematic diagram of the hydrogel bioelectronics. The device features three main components: electrical controlled drug release hydrogel along with electrostimulation; drug refillable chamber that can support continuous use for over 20 days; programmable dosing rhythm under control of a flexible circuit with bluetooth. (B) Exploded layered view of the device, including flexible circuit, polydimethylsiloxane (PDMS) membrane, drug chamber, titanium mesh electrodes, and hydrogel. Reprinted with permission from Ref. [64].

Wearable iontophoresis devices integrate flexible substrates (e.g., polyimide, and MXene) and stretchable electrodes to ensure mechanical compatibility with dynamic biological tissues. A study conducted by Kamoto et al. [63] developed a wearable iontophoresis device comprising a flexible polyimide substrate and disposable stretchable electrodes for the transdermal delivery of anticancer vaccines. While conventional hydrogel dressings remain foundational in wound care, their static polymer networks impose suboptimal drug-loading capacities and necessitate repetitive dressing changes. These limitations synergistically induce iatrogenic tissue trauma during replacement procedures and generate erratic drug release kinetics. To overcome these limitations, Du's research group engineered a bioelectronic hydrogel system (BHS) featuring *in situ* drug replenishment via a modular architecture that preserves device integrity across treatment cycles. The BHS integrates three functionally optimized layers: conductive hydrogel, a soft chassis made of polydimethylsiloxane (PDMS), and flexible circuits (Fig. 5) [64]. Besides, these devices can dynamically adjust drug delivery based on patient responses or environmental factors, offering a more tailored treatment approach. A facilitated TDDS using nanocarriers embedded in a hydrogel with reverse electrodialysis demonstrated the potential for combining iontophoresis with advanced drug delivery technologies [65]. Concurrently, innovations in self-powered systems, such as triboelectric nanogenerators (TENGs) are revolutionizing portability, given their ability to harvest energy from body movements (1.2 mW/cm^2^ output), eliminating external power dependency, enhancing portability for chronic disease management. These devices synergize with microfluidic sweat patches to enable continuous biomarker analysis, crucial for optimizing drug dosing [66]. This innovation eliminates the need for external power sources and allows for continuous, non-invasive transdermal drug delivery. A study conducted by Wu et al. [51] demonstrated the use of TENGs in a self-powered iontophoresis system, enabling both drug delivery and real-time monitoring.

Despite these promising technological advancements, iontophoretic drug-device systems face substantial regulatory hurdles due to their classification as combination products. The U.S. Food and Drug Administration (FDA) requires iontophoresis systems intended for specific drug delivery to undergo review under drug-device combination pathways, in contrast to general-purpose iontophoresis devices, which qualified for a 2016 reclassification that lowered their designation from Class III to Class II [67]. An analysis of 129 FDA decisions on combination products found that 42% were classified as drug-led, 37% as device-led, and 21% required dual-path or clarifying review, illustrating the lack of regulatory predictability. Furthermore, combination products typically require a longer average approval time (22.3 month) compared to devices (10.1 month) and drugs (14.2 month), with estimated added development costs ranging from $6 to $10 million. These regulatory barriers may delay clinical translation and increase commercialization risk, particularly for innovative self-powered or feedback-responsive systems [68].

### 5.2 Closed-loop systems: dynamic feedback and AI-driven precision

**Fig. 6:**
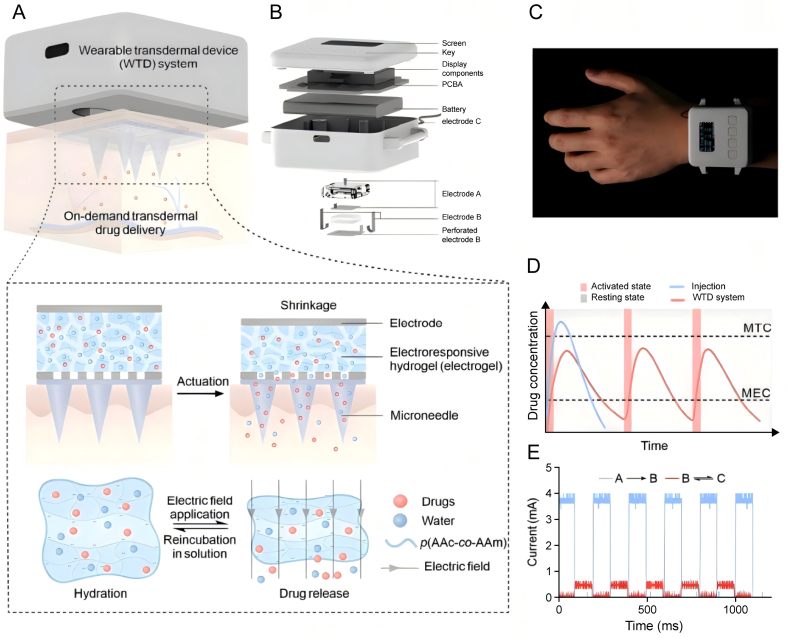
Schematic of the wearable transdermal device (WTD) system. (A) Schematic of transdermal drug delivery with the WTD system. (B) Exploded view of the corresponding subcomponents. (C) The WTD worn on the arm. (D) Schematic of drug concentration curves after transdermal drug delivery by injection or using the WTD system. (E) Typical output current between electrode pairs: A and B (blue); B and C (red). Reprinted with permission from Ref. [72]. MTC: maximum tolerated concentration; MEC: minimum effective concentration; PCBA: printed circuit board assembly.

The integration of real-time biosensing with adaptive drug release exemplifies the paradigm shift toward autonomous therapeutic systems. A randomized controlled trial (*n* = 31 participants) comparing wireless patches and wired dose controllers in treating patellar tendinopathy demonstrated equivalent efficacy in reducing pain and improving function. Participants (24.5 ± 5.9 years, 22 males) received six 80 mA/min iontophoresis treatments with 4% dexamethasone sodium phosphate. Both active treatment groups showed clinically significant improvements in the Kujala Anterior Knee Pain Scale (*P* > 0.05 between groups), with greater pain relief during functional tasks (sit-to-stand tests) compared to sham controls (*P* = 0.042). Notably, the wireless system maintained therapeutic equivalence while enabling patients to perform daily activities, thereby validating its ambulatory feasibility with a 24-h battery life and automatic dose termination [69,70]. Interestingly, the California Institute of Technology's aptamer-based nanosensor detected sweat estradiol at 0.14 pM, correlating strongly with blood levels [71], while AI algorithms integrated into Wanget al.'s wearable transdermal device (WTD) (Fig. 6) [72] dynamically adjusted insulin/glucagon ratios, maintaining glucose control within about 10% error in diabetic models. Such minimally invasive, on-demand drug delivery systems integrate drug-loaded electrogels for electric field-controlled high-dose release, utilizing dissolvable microneedles to bypass the skin barrier by accelerating hydration and enhancing transdermal transport via iontophoresis, thereby overcoming limitations imposed by the skin's interstitial fluid. The success of this system mirrors that of artificial pancreas technologies, where hybrid closed-loop systems reduced hypoglycemia by 40% in clinical trials through continuous glucose monitoring and algorithm-driven insulin delivery. In the field of oncology, flexible iontophoresis patches delivering programmed cell death protein 1 (PD-1) inhibitors combined with temperature sensors achieved 70% tumor reduction in preclinical models, demonstrating localized efficacy with minimized systemic toxicity [73]. Further advancing personalized medicine, AI-driven multi-modal data fusion is being used to optimize dosing. A system developed at Tianjin University integrates electrocardiogram (ECG) data with interstitial lithium ion monitoring to predict bipolar disorder episodes, significantly reducing dosage fluctuations. This is achieved through a wearable monitoring node equipped with three electrodes, capable of collecting real-time ECG signals with satisfactory accuracy. The gathered data are transmitted to the Internet-of-Things cloud using Wi-Fi, leveraging its high data rates and wide coverage areas [74]. For the wearable iontophoresis device, the electrical control circuit is crafted on a flexible polyimide substrate using photolithography and the stretchable electrodes, combined with the flexible polyimide substrate, are designed to be disposable. The device allows for the real-time setting and monitoring of the current applied to the skin directly through a smartphone.

### 5.3 Integrating iontophoresis and electrochemical sensing for personalized drug delivery with wearable devices

**Fig. 7:**
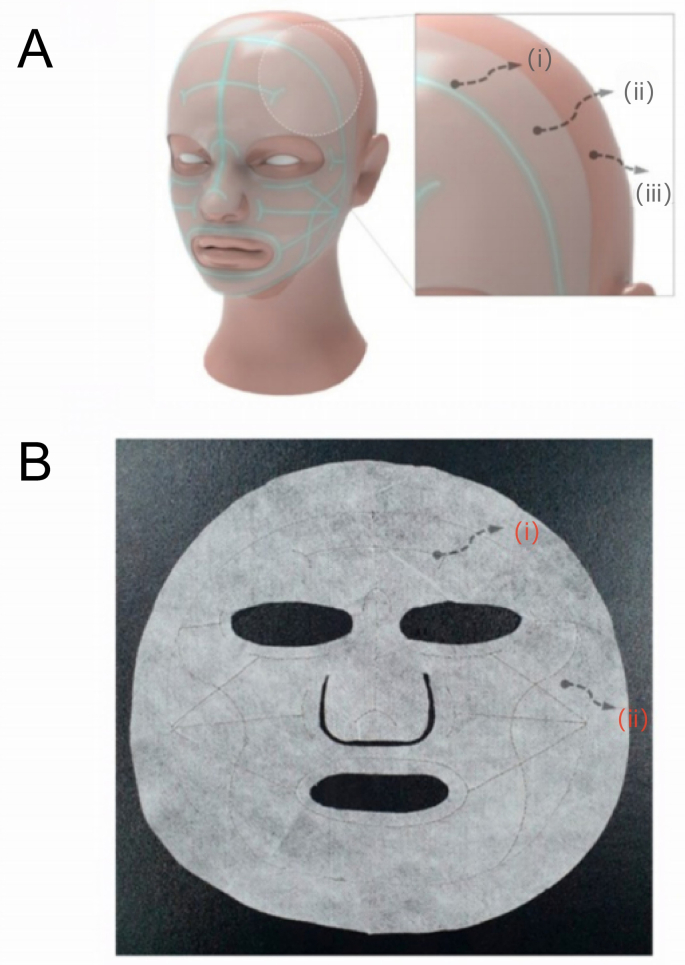
Prototype and operational schematic of an electric facial mask with Ag yarn electrodes. (A) Application of a facial mask with an embedded electrode. (B) Produced electric facial mask. The arrows in the enlarged part indicate the (i) Ag yarn, (ii) nonwoven felt, and (iii) facial skin. Reprinted with permission from Ref. [75].

One of the key challenges in managing chronic conditions is ensuring patient compliance with medication regimens. To address the limitations of traditional iontophoresis devices, Bok et al. [75] developed a portable, flexible, and stable electric facial mask system (Fig. 7) [75], integrating iontophoresis to enhance and evaluate drug penetration into skin tissue. Wearable and portable iontophoresis devices, which can be operated hands-free, offer greater convenience and comfort for patients. These devices facilitate self-administration, reducing the need for complex or invasive procedures. The development of portable iontophoresis devices demonstrated effective drug delivery with ease of operation, contributing to better adherence and patient satisfaction. The study conducted by Chen et al. [76] presents the development of an integrated portable electrochemical sensor combining gold nanoparticles (AuNPs) and MXene-modified screen-printed electrodes with a low-power electronic system for point-of-care monitoring of serum biomarkers. The AuNPs/MXene nanocomposite significantly enhanced electrochemical performance by increasing active sites, conductivity, and catalytic activity, achieving detection limits of 1.12 μM for uric acid (UA) and 1.11 μM for dopamine through differential pulse voltammetry. The sensor demonstrated excellent selectivity against common serum interferents and showed strong correlation with conventional biochemical systems in clinical UA detection. Expanded applications included the quantification of cystatin C (Cys C) for predicting the risk of gestational diabetes mellitus (GDM), revealing elevated Cys C levels in GDM patients through a papain-functionalized detection mechanism. Key innovations include automated Screen-Printed Electrode fabrication (0.05 mm precision), a miniaturized Bluetooth-enabled potentiostat, and 11-fold cost reduction compared to commercial electrodes, positioning this platform as a transformative solution for decentralized healthcare monitoring and wearable diagnostics [76].

## 6 Pathogenesis and iontophoresis-assisted therapeutic strategies in inflammatory dermatoses

### 6.1 Psoriasis

**Fig. 8:**
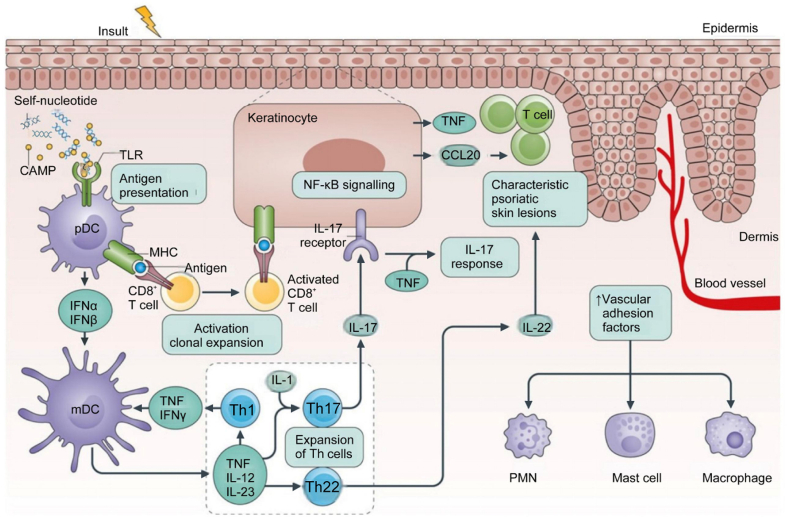
Mechanisms of psoriasis. Reprinted with permission from Ref. [78]. CAMP: cathelicidin antimicrobial peptide; pDC: plasmacytoid dendritic cell; IFN-α/β: interferon-alpha/beta; IFNγ: interferon-gamma; mDC: myeloid dendritic cell; MHC: major histocompatibility complex; CD8^+^ T cell: cluster of differentiation 8 positive T cell; TNF: tumor necrosis factor; Th1: T helper 1 cell; IL-1: interleukin-1; NF-κB: nuclear factor kappa-light-chain-enhancer of activated B cells; CCL20: C-C motif chemokine ligand 20; TLR: toll-like receptor; IL-17: interleukin-17; IL-22: interleukin-22; Th17: T helper 17 cell; Th22: T helper 22 cell; PMN: polymorphonuclear neutrophil.

Psoriasis is a chronic immune-mediated inflammatory skin disease characterized by keratinocyte hyperproliferation and immune cell infiltration, affecting all ages with no sex bias. Its severity ranges from scattered red, scaly plaques to widespread skin or joint involvement, often worsening with age or waxing and waning. The global prevalence of psoriasis is approximately 2%–3% [77]. The pathogenesis is multifactorial, involving a complex interplay of genetic predisposition, environmental triggers, and immune dysregulation (Fig. 8) [78]. External triggers, such as trauma and infections, can release self-nucleotides in genetically susceptible individuals, which complex with antimicrobial peptides and activate plasmacytoid dendritic cells (pDCs). These pDCs activate cluster of differentiation 8 positive T lymphocytes (CD8^+^ T cells), which migrate to the epidermis, releasing cytokines that fuel inflammation and keratinocyte proliferation. Moreover, pDCs secrete interferons (IFNs), activating myeloid dendritic cells (mDCs) and T helper cells (Th1, Th17, Th22), which produce cytokines like IL-17 and IL-22. Angiogenesis and vascular changes also contribute to characteristic psoriatic plaques [79,80]. However, current psoriasis therapies face significant challenges, including systemic toxicity, long-term TCS-induced skin thinning, accessibility limitations of injectable biologics like adalimumab despite efficacy, and phototherapy side effects like dryness and ultraviolet (UV) radiation risk [81]. Table 2 [[82], [83], [84], [85], [86], [87], [88], [89], [90], [91]] summarizes various drugs used in the treatment of psoriasis. Iontophoresis offers a promising approach to overcome these limitations by electrically enhancing topical drug absorption, thereby enabling targeted delivery to localized plaques. This approach reduces systemic administration needs, minimizes toxicity, lowers required doses while improving efficacy, and enhances patient compliance via localized treatment with fewer systemic effects. For instance, the use of anti-IL-17 agents, which have demonstrated significant efficacy in clinical trials, could be optimized through iontophoretic delivery in the future, allowing for localized action at the site of inflammation [92].

**Table 2:** Overview of psoriasis treatment techniques and drugs.

Drug	Indication	Mechanism of action	Iontophoresis applicability	Advantages	Refs.
MTX	Moderate-to-severe psoriasis and psoriatic arthritis	Inhibiting dihydrofolate reductase, reducing T-cell activity and proliferation	Suitable for iontophoresis. At physiological pH, MTX is water-soluble and negatively charged, enabling electromigration-driven delivery into skin layers	Reducing systemic toxicity; targeting lesions locally	[82,83]
Cyclosporine	Severe psoriasis	Suppressing T-cell activity and reducing inflammation by inhibiting calcineurin	Potential for iontophoresis. As a hydrophobic macromolecule, electroosmotic flow may facilitate transdermal penetration, though optimization is needed	Improving localized immunosuppression, reducing systemic side effects	[82]
TCSs	Mild-to-moderate psoriasis	Reducing inflammation by inhibiting multiple inflammatory pathways	Potential for iontophoresis. Their lipophilic nature can be leveraged with EO to enhance transdermal penetration into inflamed psoriatic skin	Enhanced penetration can improve efficacy for mild or moderate psoriasis	[84]
Biologics (adalimumab, etanercept, ustekinumab)	Moderate-to-severe psoriasis	Targeting specific immune pathways, such as TNF-α, IL-12, and IL-23	Challenging for direct iontophoresis due to large protein size. Novel strategies (e.g., nanocarrier conjugation) can enable delivery, but clinical evidence is lacking	Localized delivery can minimize systemic immunosuppression	[85]
Crisaborole (topical PDE-4 Inhibitor)	Mild-to-moderate psoriasis	Inhibiting PDE-4, reducing inflammatory cytokines	Potential for iontophoresis. As a small molecule, electromigration or EO can enhance penetration into psoriatic lesions	Improved delivery can boost anti-inflammatory effects	[86]
Retinoids (acitretin)	Severe psoriasis	Regulating skin cell growth by activating retinoic acid receptors	Potential for iontophoresis. Their lipophilic structure can be complemented by electroosmotic flow to improve transdermal delivery	Enhanced penetration can better regulate keratinocyte growth	[87]
Phototherapy	Moderate-to-severe psoriasis	Using UV light to slow down skin cell turnover	Not applicable	Not applicable	[88]
JAK Inhibitors (tofacitinib)	Moderate-to-severe psoriasis	Inhibiting JAK pathways, reducing inflammatory cytokines	Potential for iontophoresis. Small molecular size allows electromigration or EO to boost localized delivery to psoriatic skin	Localized delivery can reduce systemic off-target effects	[89]
Vitamin D analogues (calcipotriol)	Mild-to-moderate psoriasis	Regulating skin cell growth and differentiation	Potential for iontophoresis. Small molecular structure enables electromigration or EO to improve transdermal penetration	Enhanced delivery can better regulate skin cell differentiation	[90]
Coal tar	Mild-to-moderate psoriasis	Reducing scaling and itching by slowing skin cell turnover	Potential for iontophoresis. While components are complex, iontophoresis can enhance delivery of soluble/charged constituents to psoriatic skin	Improved delivery can enhance anti-scaling/anti-itching effects	[90,91]

MTX: methotrexate; EO: electroosmosis; TCSs: topical corticosteroids; TNF-α: tumor necrosis facto-α; IL-12: interleukin-12; PDE-4: phosphodiesterase-4; UV: ultraviolet; JAK: Janus kinase.

Methotrexate (MTX) is a commonly indicated for psoriasis therapy, yet systemic toxicity limits its oral or injectable use. *In vitro* studies by Alvarez-Figueroa et al. [93] have demonstrated that iontophoresis could significantly enhance MTX skin penetration compared to passive diffusion (*P* < 0.05), with 10-h cumulative delivery consistently higher across varied conditions despite modest sample sizes (*n* = 6 replicates per group). Clinical efficacy, however, was contingent on specific parameters. Mechanistic findings from the study indicated that higher current density (0.5 mA/cm^2^) directly enhanced MTX transdermal flux, with the amount of MTX delivered increasing with current density and showing statistically significant differences under specific conditions. In a study conducted by Chandrappa et al. [10], which compared MTX iontophoresis with 0.05% clobetasol propionate ointment, a minimum sample size of 25 participants per group was determined. This sample size calculation was based on attaining 80% statistical power while accounting for potential participant attrition. Weekly assessments were conducted in a consistent manner. The results indicated that the proportion of satisfactory improvement was 32% in one group and 48% in the other group (*P* = 0.25). These findings lend support to the reliability of the observed non-significant difference between the two treatment modalities. A similar study conducted by Haseena et al. [11] demonstrated that among the 28 patients who completed the study, 20 patients showed more than 50% improvement after 6 treatment sessions of iontophoretic delivery of MTX. The positive outcome was attributed to higher average current intensity (7.5 mA) as compared to 4.5 mA in Chandrappa et al.'s study [10].

For nail psoriasis, achieving therapeutic drug concentrations at inflammatory sites is challenging due to anatomical barriers. However, iontophoresis combined with triamcinolone acetonide could enhance penetration and reduce treatment frequency. Saki et al. [14] conducted a bilateral controlled trial in 16 patients, using a self-controlled design: one hand received monthly triamcinolone acetonide iontophoresis (TI: 4 mA for 20 min) and the other daily topical calcipotriol/betamethasone dipropionate (CB) for 6 month. Efficacy was assessed via the nail psoriasis severity index (NAPSI). No significant differences were found between TI and CB in reducing nail bed scores (*P* = 0.356), matrix scores (*P* = 0.137), or total NAPSI (*P* = 0.098), confirming comparable efficacy. The modest sample size (16 completers) was justified by the self-controlled design, with appropriate statistical tests showing significant within-group improvements (TI reduced NAPSI by 8.69 ± 4.05, *P* < 0.001). Notably, TI had no reported side effects and offered better compliance via monthly sessions versus daily CB, although larger trials are needed to confirm generalizability. The efficacy of iontophoresis can be further enhanced when combined with advanced topical formulations, such as microemulsions or hydrogels. These formulations improve drug stability and bioavailability, leading to enhanced delivery through the skin. Fukuta et al. [13] investigated the iontophoretic delivery of biologics in a non-invasive way for the treatment of psoriasis. Iontophoresis successfully delivered approximately 80% of large, hydrophilic fluorescein isothiocyanate-labeled immunoglobulin G (FITC-labeled IgG) antibodies into the skin tissue of hairless rats, showing efficient transdermal delivery and penetration into the epidermis and dermis, which is not achieved by passive diffusion. Next, they investigated the intradermal distribution of FITC-IgG 1 under iontophoresis assistance. The fluorescence derived from FITC-IgG was mainly detected by iontophoresis-mediated administration of the antibody. These results collectively revealed that iontophoresis can be effectively applied for intradermal delivery of biologics, even in inflamed skin.

### 6.2 AD

**Fig. 9:**
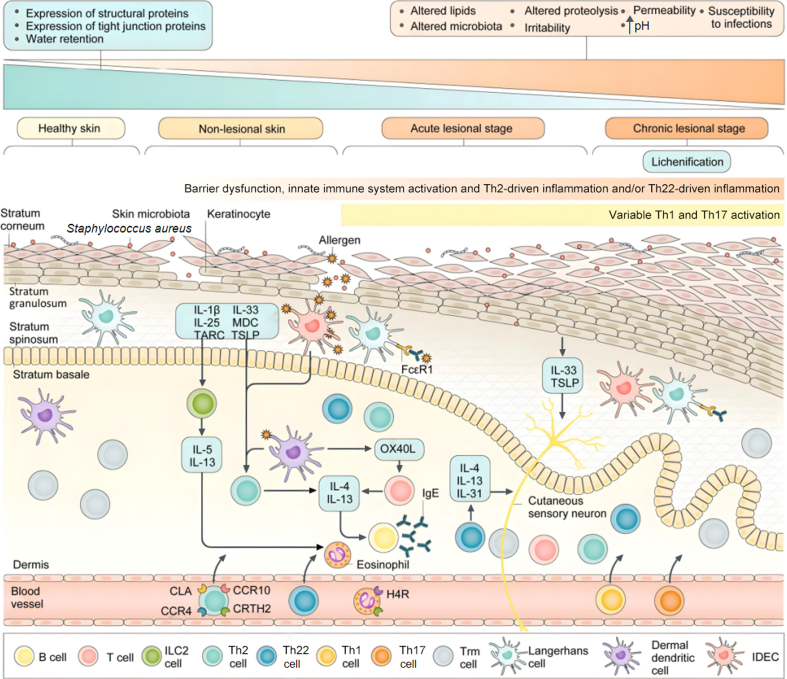
Mechanisms of atopic dermatitis (AD). Reprinted with permission from Ref. [95]. Th1: T helper 1 cell; IL-4: interleukin-4; TSLP: thymic stromal lymphopoietin; TARC: thymus and activation-regulated chemokine; CLA: cutaneous lymphocyte-associated antigen; CCR: C-C motif chemokine receptor; OX40L: OX40 ligand; Trm: tissue-resident memory T cell; IDEC: inflammatory dendritic epidermal cell; H4R: histamine H4 receptor; IL-25: interleukin-25; IL-33: interleukin-33; IL-31: interleukin-31; FcεRI: high-affinity IgE receptor; ILC2: type 2 innate lymphoid cell; IgE: immunoglobulin E; CRTH2: chemoattractant receptor-homologous molecule expressed on Th2 cells; CCR 4/10: C-C chemokine receptor type 4/10.

AD is a chronic inflammatory skin condition characterized by immune dysregulation, genetic predisposition, and epidermal barrier dysfunction, leading to intense itching and eczematous lesions. Barrier disruption prompts keratinocytes to release cytokines (IL-1β, IL-33) [94], activating Th2 cells and promoting IgE-mediated inflammation(Fig. 9) [95]. In addition, dendritic cells capture allergens and damaged skin molecules, presenting them to T cells, leading to allergic and immune responses. CD8^+^ T cells and Th2 cells infiltrate the skin, further increasing inflammation and itching [96]. Environmental factors such as microbiota imbalance and genetic variants also contribute to disease susceptibility. These pathophysiological features, particularly the impaired skin barrier, limit the efficacy of topical treatments [97]. Table 3 [[98], [99], [100], [101], [102], [103], [104], [105], [106], [107], [108], [109]] summarizes various drugs used in the treatment of AD. Current AD treatments face challenges, including side effects, limited skin penetration, and poor patient compliance. TCSs, while effective, can induce skin thinning, limiting long-term use. TCIs (tacrolimus, pimecrolimus) avoid skin atrophy but may cause irritation and have a slower onset of action [99]. Dupilumab, although highly effective for severe AD, is costly and requires regular injections [100]. Non-steroid options like crisaborole are less irritating but less effective for severe cases. Systemic treatments like cyclosporine necessitate close toxicity monitoring, restricting their long-term use [101]. Iontophoresis holds promise by enhancing the penetration of such molecules, enabling the targeted delivery of drugs directly through the SC to the epidermis. Kigasawa et al. [15] evaluated small interfering RNA (siRNA) delivery via iontophoresis in an AD rat model using 16 Brown Norway rats divided into four groups (*n* = 4 each): non-treated, ovalbumin (OVA)-treated, OVA + anti-luciferase siRNA (control), and OVA + anti-IL-10 siRNA. Iontophoresis (0.3 mA/cm^2^ for 1 h) successfully enabled epidermal penetration of Cy3-labeled siRNA, as confirmed by confocal microscopy, whereas passive delivery resulted in siRNA remaining on the skin surface. Treatment with iontophoretic anti-IL-10 siRNA significantly reduced IL-10 messenger RNA (mRNA) expression by 73% (*P* = 0.024, Student's *t*-test). No tissue damage or off-target effects on housekeeping genes were observed. However, the study's limitations include a small sample size, short (12-h) efficacy, and the use of a rodent model. Next, they investigated whether siRNA delivered via iontophoresis could alter the level of expression of a target gene involved in AD, finding that target gene expression was significantly reduced by RNA interference (RNAi) [15]. Iontophoretic delivery has also been explored for oligonucleotide therapies targeting specific inflammatory pathways in AD. For instance, antisense oligonucleotides targeting IL-10 were effectively delivered via iontophoresis in mouse models, reducing both IL-10 levels and the severity of AD symptoms [110]. This suggests a potential immunotherapeutic application for iontophoresis in modulating key cytokines involved in AD pathogenesis. Recent research has highlighted the efficacy of iontophoresis in conjunction with specific topical formulations for treating AD. For instance, a study by Eladl et al. [111] compared the effects of photochemotherapy and tap water iontophoresis on children with AD. They noted that some sensations were more pronounced during pulsed tap water iontophoresis for erosive skin changes compared to passive diffusion, necessitating the covering of erosions with petrolatum to lessen these sensations.Their findings revealed that combination therapy significantly improved clinical outcomes compared to medical therapy alone. While iontophoresis can be helpful in cases of atopic eczema, it did not significantly outperform other established treatments like corticosteroids or psoralen plus ultraviolet A (PUVA) alone in a study conducted by Tupker et al. [20]. In this study, 48 patients with chronic moderate-to-severe foot eczema were randomly divided into 3 groups: the local bath-PUVA with iontophoresis group, the only PUVA group and the corticosteroid group treated with fluticasone. However, in a randomized observer-blinded trial, iontophoresis combined with local bath-PUVA did not demonstrate superior efficacy compared with PUVA alone or topical corticosteroid therapy in patients with chronic foot eczema.

**Table 3:** Overview of atopic dermatitis (AD) treatment techniques and drugs.

Drug	Indication	Mechanism of action	Iontophoresis applicability	Advantages	Refs.
TCSs	Mild-to-moderate flare-ups	Anti-inflammatory; suppressing cytokines and immune response	Potential for iontophoresis. Lipophilicity can be paired with EO to enhance transdermal penetration into AD lesions	Enhanced penetration can improve anti-inflammatory efficacy	[98,102]
TCIs (tacrolimus, pimecrolimus)	Moderate-to-severe AD; maintenance therapy	Inhibiting T-lymphocyte activation, reducing inflammation	Potential for iontophoresis. Small molecular size allows electromigration or EO to improve localized delivery to AD skin	Localized delivery can reduce systemic exposure	[99]
Dupilumab (biologic)	Moderate-to-severe AD in adults and children	Blocking IL-4/IL-13 pathways, reducing Th2-mediated inflammation	Challenging for direct iontophoresis due to large antibody size. Nanocarrier-based strategies could enable delivery, though clinical data are absent	Localized delivery can target IL-4/IL-13 pathways in skin	[100,103]
Crisaborole (topical PDE-4 Inhibitor)	Mild-to-moderate AD	Inhibiting PDE-4, reducing inflammation	Potential for iontophoresis. Small molecular structure supports electromigration or EO to boost anti-inflammatory effects in AD	Improved delivery can boost anti-inflammatory effects	[104]
Cyclosporine	Severe, refractory AD	Immunosuppressant; inhibiting T-cell activation	Potential for iontophoresis. Hydrophobic nature can be addressed by electroosmotic flow to enhance transdermal penetration, reducing systemic exposure	Localized delivery can reduce systemic toxicity	[101,105]
Tacrolimus (topical)	Moderate-to-severe AD	Calcineurin inhibitor; reduceing T-cell activation and inflammation	Potential for iontophoresis. Small molecular size enables electromigration or EO to improve calcineurin inhibition in AD lesions	Enhanced penetration can improve calcineurin inhibition	[106]
Dupilumab and TCSs	Moderate-to-severe AD, especially for patients who cannot tolerate ciclosporin	IL-4/IL-13 inhibition and inflammation suppression	Potential for iontophoresis. The combination could leverage enhanced delivery of both agents (e.g., TCS via EO, dupilumab via optimized strategies) to target AD pathogenesis	Synergistic targeting of IL-4/IL-13 and inflammation	[107]
Sodium cromoglicate	AD in children (ages 2–12)	Mast cell stabilizer; reducing allergic inflammation	Potential for iontophoresis. Water solubility and charge allow electromigration to improve localized mast cell stabilization in pediatric AD	Localized delivery can better stabilize mast cells in pediatric AD	[108]
Pimecrolimus (topical)	Mild-to-moderate AD	Inhibiting calcineurin, reducing T-cell activation and inflammation	Potential for iontophoresis. Small molecular size supports electromigration or EO to enhance T-cell inhibition in AD skin	Enhanced penetration can improve T-cell inhibition	[98]
JAK Inhibitors (Ruxolitinib, Upadacitinib)	Moderate-to-severe AD	Inhibiting JAK pathways, reducing inflammatory cytokine signaling	Potential for iontophoresis. Small molecular structure enables electromigration or EO to reduce systemic off-target effects in AD	Localized delivery can reduce systemic JAK pathway inhibition	[109]

TCSs: topical corticosteroids; EO: electroosmosis; TCIs: topical calcineurin inhibitors; IL-4: interleukin-4; Th2: T helper 2; PDE-4: phosphodiesterase-4; JAK: Janus kinase.

### 6.3 Acne vulgaris

**Fig. 10:**
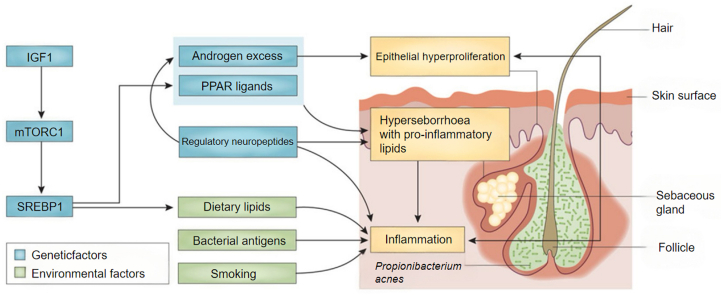
Tangled network of four core events in acne formation. Reprinted with permission from Ref. [113]. IGF-1: insulin-like growth factor-1; mTORC1: mechanistic target of rapamycin complex 1; SREBP: sterol regulatory element-binding protein; PPAR: peroxisome proliferator-activated receptor.

Acne vulgaris is a chronic inflammatory skin disorder that mainly targets the pilosebaceous units, which include hair follicles and sebaceous glands [112]. The pathogenesis of acne involves a complex interplay of four main factors: follicular hyperkeratinization, propionibacterium acnes activity within the follicle, and inflammation. These are influenced by hormonal, immune, and environmental factors (Fig. 10) [113], resulting in the formation of comedones, papules, pustules, nodules, and cysts. Hyperseborrhoea, largely driven by androgens and insulin-like growth factor 1 (IGF-1), combines with hyperkeratinization to clog pores and form comedones [112,114]. This anaerobic environment supports *Cutibacterium acnes* growth, which in turn stimulates immune responses and releases pro-inflammatory cytokines like TNF and IL-1β, escalating inflammation. Additional contributors include lipid mediators, neuropeptides, and monounsaturated fatty acids (MUFAs), which modulate sebum production and inflammation [113,115]. Acne vulgaris has multifaceted causes and can be worsened by atrophic scars and postinflammatory hyperpigmentation (PIH) [116]. Acne scars, a prevalent complication of acne vulgaris, represent persisting impairments to patient quality of life. The etiologies of atrophic scars in acne remain ambiguous, making this complication challenging to treat. Inflamed papules may appear alongside erosions overlying the inflammatory lesions. These larger, deeper lesions can rupture the overlying skin, leading to hemorrhagic crusts [117]. Table 4 [[118], [119], [120], [121], [122], [123], [124], [125], [126], [127]] summarizes various drugs used in the treatment of acne vulgaris. However, acne treatments face several challenges. Long-term antibiotic use can lead to resistance, reducing effectiveness [118]. Oral isotretinoin, while effective, may cause serious side effects such as liver toxicity and depression, requiring close monitoring [119]. Topical agents such as retinoids and benzoyl peroxide often cause irritation and dryness, affecting adherence [120]. Besides, hormonal therapies are limited to female patients and carry risks such as blood clots. Iontophoresis provides a promising solution by enhancing transdermal drug delivery, bypassing the gastrointestinal tract, and minimizing systemic side effects. This method uses a small electric current to drive charged drugs across the skin, improving penetration and absorption without causing irritation or resistance [128]. It also allows controlled and programmable delivery, ensuring that drugs are administered in a consistent and effective manner. By enhancing the absorption of topical agents, iontophoresis can reduce the need for high systemic doses, thereby helping to overcome many of the challenges faced in acne treatments. Kurokawa et al. [116] examined the use of chemical peeling (CP) with glycolic acid and iontophoresis with ascorbyl 2-phosphate 6-palmitate (APPS) and Dextro-Levo, racemic (DL)-α-tocopherol phosphate to treat PIH, erosions with inflamed red papules, and non-inflamed atrophic scars in 31 patients with acne vulgaris. The patients were treated with CP using 20% GA at pH 3.2, followed by iontophoresis with either a Moisture Gel (contains vitamin A, vitamin C, vitamin E, vitamin B5 and β-carotene or APPS and DL-α-tocopherol phosphate. Their results illustrated that treatment with CP and subsequent iontophoresis remarkably improved PIH and erosion with inflamed red papules in most cases [116]. Another study conducted by Schmidt et al. [129] investigated tretinoin-iontophoresis for the treatment of acne scars. Iontophoresis with tretinoin gel was performed twice weekly for 20 min over a period of 3 month. They found a decrease in scar depth in 94% of patients. Two patients showed no decrease in scarring according to the subjective patient opinions and investigator's clinical evaluations (Table 5) [129]. After an average of 9 weeks, subjective assessments of the skin indicated improvements in firmness in 47% of participants and a reduction in pore size in 55%. However, skin moisture levels showed no consistent trend during the treatment period, with increases observed in 16% of cases and decreases in 38%. Overall, the combination of iontophoresis with topical formulations improves the therapeutic efficacy of acne treatments, reducing the severity of acne lesions, hampering new breakouts, and accelerating skin healing, while allowing for better drug delivery without increasing the dosage.

**Table 4:** Overview of acne vulgaris treatment drugs.

Drug	Indication	Mechanism of action	Iontophoresis applicability	Advantages	Refs.
Topical retinoids (tretinoin, adapalene, tazarotene, trifarotene)	Mild-to-moderate acne; comedonal and inflammatory lesions	Promoting cell turnover, and preventing follicular plugging	Potential for iontophoresis. Lipophilicity can be complemented by electroosmotic flow to improve transdermal penetration for follicular targeting	Enhanced penetration can improve cell turnover and follicular plugging	[119]
Oral isotretinoin	Severe nodular acne	Reducing sebaceous gland size and sebum production	Not applicable	Not applicable	[119,121]
Benzoyl peroxide	Mild-to-moderate acne; inflammatory acne	Antimicrobial; reducing C. acnes, and preventing antibiotic resistance	Potential for iontophoresis. Small molecular size allows electroosmosis (EO) to boost antimicrobial penetration into acne lesions	Improved delivery can boost antimicrobial effects	[118,120]
Topical antibiotics (clindamycin, erythromycin)	Mild-to-moderate inflammatory acne	Suppressing C. acnes growth, and reducing inflammation	Potential for iontophoresis. Small molecular size supports electromigration or EO to improve localized antibacterial effects, reducing systemic resistance risk	Localized delivery can reduce systemic antibiotic resistance risk	[118,122]
Oral doxycycline/minocycline	Moderate-to-severe acne; inflammatory lesions	Reducing C. acnes growth, and anti-inflammatory properties	Not applicable	Not applicable	[123]
Hormonal therapy (spironolactone, oral contraceptives)	Moderate acne in women; especially for hormonally-driven acne	Anti-androgen; reducing sebum production	Not applicable	Not applicable	[124]
Azelaic acid	Mild-to-moderate acne	Anti-microbial, anti-inflammatory properties	Potential for iontophoresis. Small molecular size and charge allow electromigration to enhance antimicrobial/anti-inflammatory effects in acne	Improved delivery can enhance antimicrobial/anti-inflammatory effects	[120,125]
Topical clascoterone	Mild-to-moderate acne	Androgen receptor blocker; reducing sebum production	Potential for iontophoresis. Small molecular structure enables electromigration or EO to improve sebum reduction in acne-prone skin	Localized delivery can better reduce sebum production	[126]
Oral zinc salts	Mild-to-moderate acne	Anti-inflammatory, and antibacterial properties	Not applicable	Not applicable	[127]
Topical dapsone	Mild-to-moderate inflammatory acne	Anti-inflammatory, anti-bacterial properties	Potential for iontophoresis. Small molecular size supports electromigration or EO to boost anti-inflammatory/antibacterial effects in acne lesions	Improved delivery can boost anti-inflammatory/antibacterial effects	[123]
Topical salicylic acid	Mild-to-moderate acne; comedonal acne	Keratolytic; promoting exfoliation, and uncloging pores	Potential for iontophoresis. Lipophilicity can be paired with electroosmotic flow to improve keratolytic penetration and pore unclogging in acne	Enhanced penetration can improve keratolytic effects and pore unclogging	[125]

**Table 5:** Clinical effect at the end of treatment. Adapted with permission from Ref. [129].

Change trend	Firmness	Scar depth	Pore size	Moisture
Increase	14/30 (47%)	0/32 (0%)	2/31 (6%)	5/32 (16%)
Equal	16/30 (53%)	2/32 (6%)	12/31 (39%)	15/32 (47%)
Decrease	0/30 (0%)	30/32 (94%)	17/31 (55%)	12/32 (37%)
*P* value	Significant increase *P* < 0.01	Significant decrease *P* < 0.001	Significant decrease *P* < 0.01	Significant decrease *P* < 0.05

## 7 Unresolved technological challenges in iontophoresis

### 7.1 Issues of delivery efficiency and uniformity

Iontophoresis enhances transdermal drug delivery by harnessing an electric field to drive charged molecules across the skin barrier into deeper tissue layers. This approach can significantly improve therapeutic outcomes by promoting drug penetration. However, significant challenges persist, largely stemming from limitations in the uniformity and efficiency of delivery, which are largely due to the heterogeneous electrical properties of skin [130]. Variations in skin resistance and uneven electric field distribution often result in over-delivery in certain regions while others receive subtherapeutic doses. These inconsistencies not only compromise therapeutic efficacy but may also lead to localized side effects. Moreover, interindividual differences in skin structure and function, such as hydration level, SC thickness, and electrical impedance, further exacerbate the variability in delivery performance. In patients with dry or oily skin, for instance, reduced hydration or excessive surface lipids can significantly diminish iontophoretic efficiency. Thus, ensuring a uniform electric field across the skin and achieving deep, targeted delivery are key technical challenges that must be addressed to optimize this technology [131]. Iontophoretic drug delivery is notably influenced by inter-patient variability, particularly due to differences in skin impedance and barrier properties. In a modeling study, Pang et al. [132] simulated three distinct skin impedance models and found that impedance values ranged from 1.2 kΩ to 5.6 kΩ, significantly affecting current flow and drug delivery rates, demonstrating up to a 4.6-fold variation in simulated iontophoretic flux between skin types. Singh et al. [133] conducted an *in vitro* study using excised human skin and found that the iontophoretic flux of phenylethylamine ranged from 2.3 to 9.8 μg/cm^2^/h depending on whether the SC was intact or tape-stripped, highlighting a 4.3-fold difference attributable to skin condition alone. Moreover, Dhote et al. [134] reported that patient-specific factors such as SC hydration could alter drug permeability by up to 3.5 times, depending on environmental humidity and anatomical site. These studies quantitatively demonstrate that skin-related physiological variability can result in significant fluctuations in iontophoretic drug delivery efficacy across individuals.

### 7.2 Electric field distribution control

Uneven electric field distribution on the skin surface can lead to localized “hotspots”, resulting in excessive drug accumulation in some areas and inadequate delivery in others. This not only reduces therapeutic uniformity but can also cause adverse effects such as local irritation or skin damage [135]. The spatial variability of skin resistance is a major factor contributing to these electric field inconsistencies. Advanced strategies have been proposed to overcome this limitation, including the development of intelligent electrode materials that can monitor and dynamically adjust current distribution in real-time. These smart electrodes are highly adaptive; they can modulate current strength and distribution in response to variations in skin impedance, environmental conditions, and physiological factors, thereby maintaining a more homogeneous electric field and balanced drug delivery [136].

### 7.3 Localized drug accumulation

Inhomogeneous electric fields in iontophoresis can lead to excessive local accumulation of the delivered agent, increasing the risk of skin inflammation, allergic reactions, or cytotoxicity, particularly if the drug fails to diffuse or distribute adequately post-delivery. In contrast, underexposed regions may exhibit reduced therapeutic efficacy [137]. Computational modeling has proven invaluable for addressing this challenge. By creating multidimensional physical models that simulate electrical resistance, conductivity, and skin thickness, researchers can visualize electric field distribution across skin layers and optimize electrode configurations accordingly [138]. These models can be personalized based on individual skin characteristics, providing a data-driven basis for tailoring treatment and improving both safety and efficacy.

### 7.4 Precision control of penetration depth

Different layers of the skin exhibit distinct structural properties and absorptive capacities. Hence, achieving precise control over the depth and rate of drug penetration is critical for effective treatment. While the SC and epidermis act as significant barriers, the dermis and subcutaneous tissues possess higher permeability. Without accurate control, drug molecules may remain trapped in the upper layers, thereby limiting therapeutic benefit. Recent research has explored the use of adjunctive physical methods, such as ultrasound and photothermal stimulation, to enhance both delivery depth and uniformity. Ultrasound temporarily disrupts the skin's structure through mechanical vibration, increasing permeability. Photothermal effects can modulate local temperature and improve electric field distribution, thereby facilitating deeper and more efficient transdermal transport. These techniques not only address field inhomogeneity but also improve the precision and efficiency of deep-layer delivery [139,140]. In summary, the issues of delivery efficiency and uniformity in iontophoresis primarily stem from electric field heterogeneity, localized drug overload, and insufficient control over delivery depth. To address these limitations, integrated solutions involving smart electrodes, computational modeling, and adjunctive physical enhancement methods are essential to improve spatial uniformity, penetration precision, and overall therapeutic outcomes.

### 7.5 Cost-effectiveness and long-term value of iontophoretic therapies

Although iontophoresis provides non-invasive, targeted drug delivery, its cost-effectiveness compared to injectable therapies remains inconsistent and highly context-dependent. A comparative cost analysis of dexamethasone iontophoresis versus intramuscular injection for musculoskeletal inflammation showed 25%–40% higher upfront costs for iontophoresis, driven largely by device costs and the need for multiple sessions [141]. Despite these higher initial costs, iontophoresis offers potential long-term savings. For instance, in a pediatric surgical setting, iontophoresis reduced procedural anxiety and eliminated the need for needles, with 88% of parents reporting satisfaction, suggesting improved treatment adherence and reduced need for sedation or monitoring. Besides, iontophoresis enables more stable drug plasma concentrations compared to injections, reducing peaks and troughs and potentially decreasing systemic side effects and follow-up costs [142]. However, no comprehensive cost-utility studies are available for iontophoresis in chronic dermatoses remains a significant gap in the literature. Current evidence does not yet justify its cost superiority over injectables in long-term dermatological management.

## 8 Conclusions and future directions

Inflammatory dermatoses encompass a group of skin disorders characterized by inflammation and immune dysregulation, leading to symptoms such as redness, swelling, itching, and scaling. These conditions often result from an abnormal immune system response and can range in severity from mild to severe. While treatments often aim to control the inflammatory pathways responsible for the immune response, which includes topical therapies and biologics, many effective options have various adverse effects. This highlights the pivotal need to develop a drug delivery approach that improves drug therapeutic effects, ensures patient compliance, and minimizes side effects. Hence, iontophoresis-assisted transdermal drug delivery has gained considerable attention. Iontophoresis-assisted transdermal drug delivery has proven to be a transformative approach for the management of inflammatory dermatoses, such as AD, psoriasis, and acne vulgaris. Its ability to enhance drug penetration through the skin's barrier, particularly the SC, addresses a critical limitation in traditional topical treatments. As discussed in this review, iontophoresis enhances the local delivery of therapeutic agents, including MTX, corticosteroids, and emerging biologics, thereby allowing for effective treatment with reduced systemic exposure and toxicity. A critical advantage of iontophoresis lies in its remarkable adaptability, enabling the transdermal delivery of a diverse spectrum of therapeutic agents, from low-molecular-weight compounds like corticosteroids to larger, more complex biological macromolecules such as monoclonal antibodies and nucleic acid-based drugs. This versatility establishes iontophoresis as an invaluable modality for targeting localized inflammatory conditions, while simultaneously mitigating the systemic side effects typically associated with conventional therapies, such as immunosuppressants or oral retinoids, which are often prescribed for severe psoriasis and AD. Furthermore, the integration of iontophoresis with complementary transdermal enhancement strategies, including microneedles, sonophoresis, and chemical permeation enhancers, has exhibited significant synergistic potential. These hybrid approaches not only facilitate the deeper penetration of otherwise challenging therapeutic molecules but also enable precise control over drug release kinetics, contributing to sustained therapeutic efficacy. In particular, the development of advanced delivery platforms, such as hydrogels and microemulsions, holds significant promise in enhancing the stability, solubility, and bioavailability of drugs administered via iontophoresis, offering a forward-looking approach to improving clinical outcomes in patients suffering from treatment-resistant dermatoses. In the specific context of wearable devices, iontophoresis offers exciting prospects for continuous, patient-friendly drug administration, particularly for chronic conditions like psoriasis and AD that require long-term management. The development of flexible, self-powered iontophoretic devices represents a significant step forward in enhancing patient compliance and expanding the application of this technology. Despite significant advances, several challenges in iontophoresis-assisted drug delivery remain unresolved and warrant further investigation. The variability in skin impedance across individuals and anatomical sites leads to inconsistent electric field distribution, which can result in uneven drug delivery. Further research is warranted to develop intelligent electrode systems and real-time monitoring to ensure uniform penetration and avoid localized over- or under-dosing. Achieving targeted delivery to specific skin layers without affecting surrounding tissues remains a technical hurdle. Innovations in computational modeling and adjunctive techniques, such as photothermal modulation, could help enhance precision. While iontophoresis is non-invasive, concerns about skin irritation, cumulative effects of repeated exposure, and the economic feasibility of widespread clinical adoption, especially for wearable devices, require rigorous long-term studies. Addressing these gaps will be essential for translating iontophoresis from promising research into a standardized clinical tool for managing inflammatory dermatoses.

## CRediT authorship contribution statement

**Zhixiong Wang:** Writing – review & editing, Writing – original draft, Visualization, Validation, Supervision, Resources, Methodology, Investigation, Formal analysis, Data curation, Conceptualization. **Xiumei Jiang:** Conceptualization. **Changzhao Jiang:** Conceptualization. **Xiaohua Tao:** Conceptualization. **Jincui Ye:** Data curation, Conceptualization.

## Declaration of competing interest

The authors declare that they have no known competing financial interests or personal relationships that could have appeared to influence the work reported in this paper.
